# Complete genome of *Bacillus zhangzhouensis* PAMC22242 isolated from biofilm in Svalbard, Norway

**DOI:** 10.1128/mra.00243-26

**Published:** 2026-07-29

**Authors:** Shriya Sahu, Byeollee Kim, Sungchul Lee, Sung Gu Lee, Jun Hyuck Lee, Tae-Jin Oh

**Affiliations:** 1 Department of Life Science and Biochemical Engineering, Graduate School SunMoon University 35022 , Asan, South Korea; 2 Genome-based BioIT Convergence Institute, Asan, South Korea; 3 Division of Computer Science and Engineering, SunMoon University 35022 , Asan, South Korea; 4 Division of Life Sciences, Korea Polar Research Institute 123591, Incheon, South Korea; 5 Department of Pharmaceutical Engineering and Biotechnology, SunMoon University 35022 , Asan, South Korea; Indiana University Bloomington, Bloomington, Indiana, USA

**Keywords:** *Bacillus zhangzhouensis*, complete genome, NRPS biosynthetic gene cluster, metal solubilize

## Abstract

The present study presents the complete genomic sequence of a novel *Bacillus zhangzhouensis* PAMC22242 isolated from biofilm at Kongsfjorden dock, Svalbard, Norway. The genome size is 3,507,368, with a 41.5% GC content, including genes putatively associated with non-ribosomal peptide synthetase biosynthesis and metal-related transport systems.

## ANNOUNCEMENT

*Bacillus* species are known for synthesizing lipopeptides, which enhance hydrophobicity, suppress plant disease, exhibit antimicrobial and anti-biofilm activity, and pathogen suppression (1), and may improve micronutrient availability and uptake (2). Siderophore secretion by *Bacillus* enhances micronutrient availability and nutrient cycling, which enhances nutrient availability by solubilizing essential components (2).

The Korea Polar Research Institute obtained the strain *B. zhangzhouensis* PAMC22242 from biofilm isolated from the Ny-Ålesund Kongsfjorden dock in Svalbard, Norway (78.923°N, 11.923°E). After serial dilution, 100 μL of the sample was plated on Zobell marine agar, and single colonies were obtained at 25°C by repeated streaking. The marine broth culture was incubated at 25°C for 48 h, and 1 mL was used for genomic DNA extraction. Cells were harvested using the DNeasy Blood & Tissue Kit (Qiagen, the Netherlands). Cultures were grown to OD_600_ ≥ 1.0 (high-density phase) before sampling per the manufacturer’s protocol. Nanopore (ONT) and Illumina used the same DNA extraction method. Illumina library preparation used the TreSeq DNA Nano Kit (Illumina, USA) without size selection, and sequencing was performed on a NovaSeq 6000 platform (2 × 150 bp paired-end). ONT libraries were prepared without size selection using the Native Barcoding Expansion Kit (EXP-NBD104) and 1D Ligation Sequencing Kit (SQK-LSK110). GridION sequencing used FLO-MIN106 (R9.4) flow cells with high-accuracy basecalling (HAC mode). Illumina yielded 15,006,794 paired-end reads (~4.5 Gb), which were quality filtered and adapter trimmed using fastp, while ONT sequencing generated 171,915 reads (*N*_50_ 10,594 bp) filtered with Filtlong (>95% retention; <1,000 bp removed), providing ~557.9× genome coverage with polypolish.

We produced a high-quality genome assembly using long-read assembly followed by polishing with both long- and short-read data, as mentioned by Wick et al. (3). The initial assembly was generated using Flye v2.9.3 (4), and Trycycler v0.5.4 (3) was used for reconciliation, overlap trimming, and genome circularization with rotation to a standard start position. Polishing steps employed Medaka v1.12.1 (5), Polypolish v0.6.0 (6), and POLCA v0.3.1 (7). Genome completeness and accuracy were evaluated with CheckM (8) and QUAST (9). tRNA genes were identified using Barrnap (10), while functional annotation relied on NCBI PGAP 6.10 (11) and AntiSMASH v8.0.4 (12). Taxonomic identity was evaluated using the EzBiocloud 16S rRNA gene database (13), identifying *B. zhangzhouensis* DW5-4(T) (JOTP01000061) as the closest match (100% 16S similarity), supported by OrthoANIu (98.99% ANI). All tools were run using default settings.

*B. zhangzhouensis* PAMC22242 possesses a compact, single circular genome of 3,707,368 bp with 41.3% GC content, and its genome assembly is complete (99.17%) with minimal contamination (0.21%). The strain encodes 4,050 protein-coding genes and 81 tRNAs with single contigs and an *N*_50_ length of 3,707,368, indicating a complete genome and metabolic ability. Notably, genome annotation of *B. zhangzhouensis* PAMC22242 using NCBI PGAP 6.10 identified genes associated with nutrient transport and elemental uptake, indicating potential for nutrient acquisition and mobilization. AntiSMASH v8.0.4 identified non-ribosomal peptide synthetase (NRPS) biosynthetic gene clusters involved in lipopeptide production (Table 1). The genome also contained genes associated with siderophore biosynthesis, suggesting potential roles in nutrient acquisition and environmental adaptation.

**TABLE 1:** AntiSMASH analysis of *Bacillus* sp. PAMC22242

Cluster ID	Type (NRPS/PKS)	From	To	Similarity to known cluster (Mibig ref.)	Similarity (%)
1	NRPS	372,576	456,283	Bovienimide A (BGC0002135.2)	77
2	NRPS	674,254	755,224	Thalassospiramide A (BGC0001876.2)	76
3	RRE	937,832	958,737	N-acylserinol (BGC0001683.3)	53
4	NI-siderophore	1,110,155	1,147,801	Woodybactin A, B, C, D (BGC0002469.2)	60
5	Betalactone	1,838,091	1,866,504	Fusarin C (BGC0001268.4)	54
6	T3PKS	1,984,822	2,025,922	Alkylpyrone (BGC0001831.3)	51
7	Terepene	2,211,054	2,231,947	Sodoferin (BGC0002283.2)	49
8	Betalactone	2,480,348	2,512,800	ε-poly-l-lysine (BGC0002174.2)	63
9	Terpene precursor	2,840,614	2,861,537	Pelgipeptin A, B, C, D (BGC0000403.5)	26
10	Others	3,368,529	3,409,950	Bacilysin (BGC0000888.5)	77

## Data Availability

The genome of *Bacillus zhangzhouensis* PAMC22242 has been submitted by the European Nucleotide Archive (ENA) under sample accession number PRJEB105563 and study accession number SAMEA1210839868. The raw sequencing reads have been deposited in the ENA under accession numbers ERR16145635 (Oxford Nanopore) and ERR17019651 (Illumina).
